# Supplementary material for: Asymmetric Electron-Hole Decoherence in Ion-Gated Epitaxial Graphene
Source: Sci Rep. 2017 Sep 21;7:12130. doi: 10.1038/s41598-017-12425-0 (PMC5608950; doi:10.1038/s41598-017-12425-0)
Supplement: Supplementary file 1 — Asymmetric Electron-Hole Decoherence in Ion-Gated Epitaxial Graphene [file 41598_2017_12425_MOESM1_ESM.pdf]

Supplementary Information

Asymmetric Electron-Hole Decoherence in Ion-Gated Epitaxial Graphene

Kil-Joon Min[1,2], Jaesung Park[1], Wan-Seop Kim[1] and Dong-Hun Chae[1,2]

[1]Center for Electricity and Magnetism, Korea Research Institute of Standards and Science, Daejeon 34113, Republic of Korea

[2]Nano Science Major, University of Science and Technology, Daejeon, 34113, Republic of Korea

Correspondence and requests for materials should be addressed to D.-H.C.

(email: dhchae@kriss.re.kr)

SI1. AFM and Raman maps of epitaxial graphene on SiC substrate

SI2. Magnetotransport measurement of pristine epitaxial graphene

SI3. Temperature dependences of the carrier density and mobility

SI4. Determination of the decoherence rate through a weak localization analysis

SI5. Asymmetric electron-hole decoherence: reproduced results

SI6. Analysis of the temperature dependence of the decoherence rate

SI1. AFM and Raman maps of epitaxial graphene on SiC substrate

The topographic atomic force microscopy (AFM) image here shows a surface structure with uniform terraces. The two selected height profiles provide the detailed information about the height (almost 0.5 and 0.75 nm, corresponding to two or three SiC bilayers) and the width (below 1 μm) of these terraces, indicating a smooth step structure without giant step bunching[1] (Figure S1). Raman spectroscopy was used to determine the number of layers of epitaxial graphene on SiC substrate[2]. Supplementary Figure S2 displays the Raman mapping of 2D-FWHM and the distributions of the 2D-peak position and 2D-FWHM. FWHM with a mean value at 36.7 cm^{-1} and a peak position at 2723 cm^{-1} with a narrow distribution clearly reveal the formation of monolayer graphene. Approximately 97% of the sample area is covered with monolayer graphene, as calculated from the 2D-FWHM mapping image depicted in Figure S2.

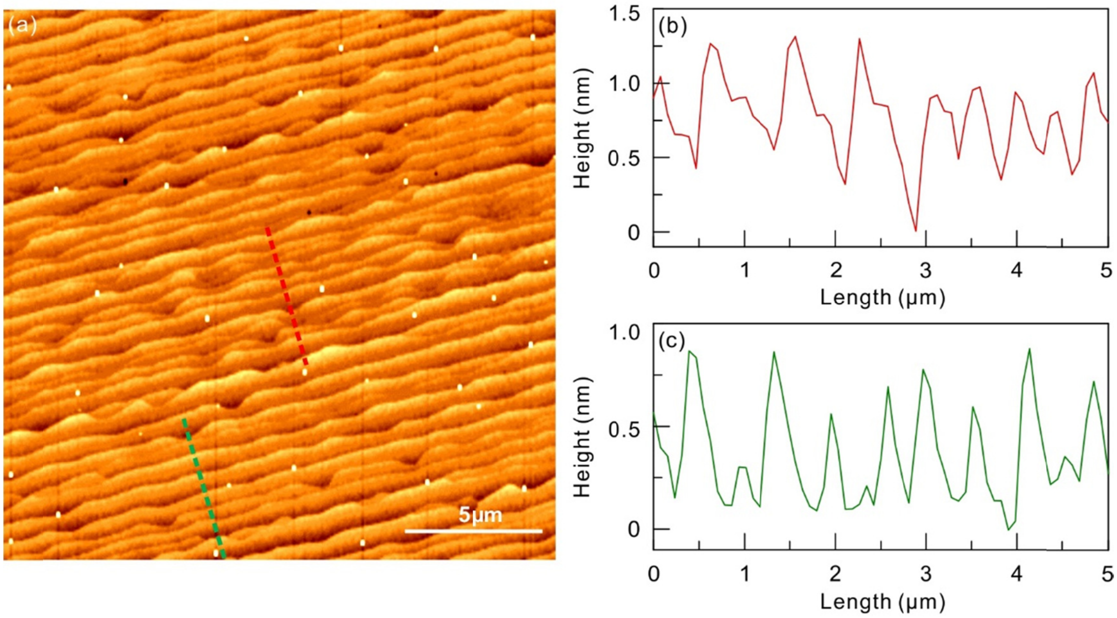

Figure S1. (a)AFM map of epitaxial graphene. (b) and (c) height profiles from red and green lines selected in (a).

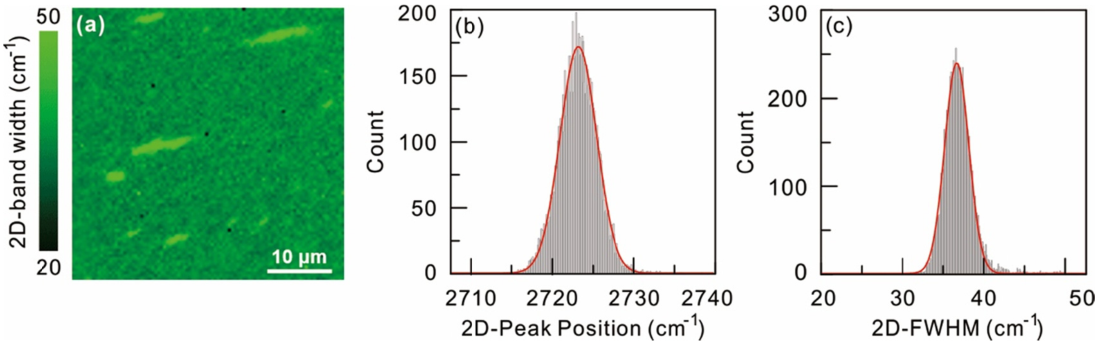

Figure S2. Monolayer coverage of graphene layer on SiC substrate. (a) Raman map of the 2D-peak full width at half maximum (FWHM). (b) and (c) Distributions of the 2D-peak position and the 2D-FWHM obtained from the mapping area (a). Red curves are Gaussian functions.

SI2. Magnetotransport measurement of pristine epitaxial graphene

We performed magnetotransport measurement of a pristine epitaxial graphene Hall bar without an ionic liquid. For comparison, a Hall bar device was fabricated on the same SiC substrate together with the studied device in the main text. Figure S3 shows an image of device (inset) and the magnetoresistance of the pristine epitaxial graphene. We observed weak localization below 1 T and Shubnikov-de Haas oscillation above about 10 T. The electron carrier density and mobility extracted from the Hall measurement are 7.90×10^{12} cm^{-2} and 900 cm^2/Vs, respectively. The phase coherence time as determined from an analysis described in SI4 is about 3.5 ps.

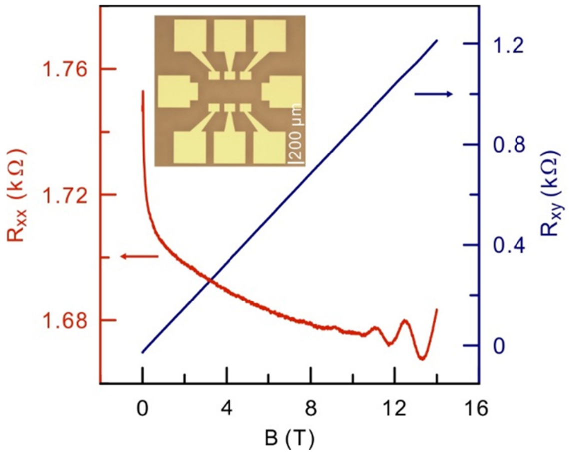

Figure S3. Magnetotransport measurement of pristine epitaxial graphene. **I**nset depicts an optical image of the device.

SI3. Temperature dependences of the carrier density and mobility

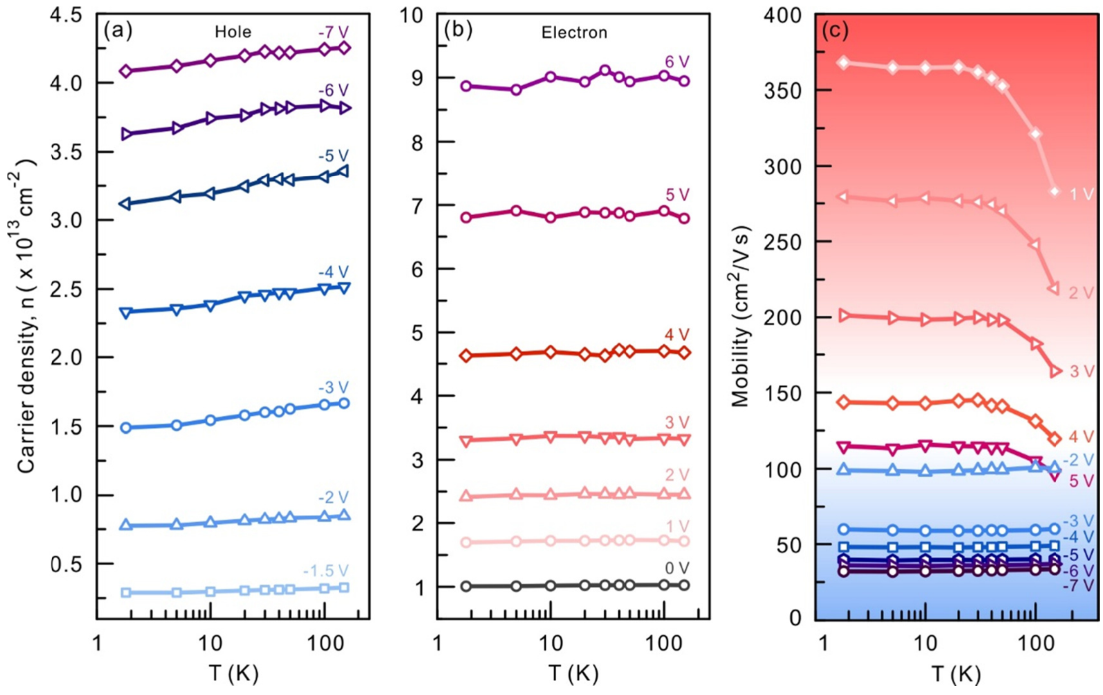

Figure S4. Temperature dependences of the carrier density (a), (b) and mobility (c) as measured in the device exhibited in the main text.

SI4. Determination of the decoherence rate through a weak localization analysis

We employ the weak localization model for graphene developed by McCann *et al.*[3] to determine the decoherence rate, $1/\tau_{\varphi}$;

$$\frac{\Delta\rho}{\rho^2} = -\frac{e^2}{\pi h}\left[F\left(\frac{B}{B_{\varphi}}\right) - F\left(\frac{B}{B_{\varphi}+2B_i}\right) - 2F\left(\frac{B}{B_{\varphi}+B_*}\right)\right] \quad (1)$$

$$F(z) = \ln z + \psi\left(\frac{1}{2}+\frac{1}{z}\right), B_{\varphi,i,*} = \frac{\hbar}{4De}\tau_{\varphi,i,*}^{-1} \text{ , } \Delta\rho\,(\mathrm{B}) \equiv \rho(\mathrm{B}) - \rho(0)$$

Here, ψ(x) is the digamma function. For this analysis, *Mathematica* was used to find optimized values of the fitting variables. With the constraint that the intervalley scattering time is shorter than the decoherence time ($\tau_i < \tau_{\varphi}; B_{\varphi} < B_i$), leading to conventional weak localization, we can obtain a convergence of B_{φ} and evaluate its reliability according to the standard error. From the determined values of B, it is possible to calculate the corresponding τ with the diffusion constant (*D*), $D = \frac{v_F l}{2}$. $l = \frac{h}{2e^2\rho\sqrt{\pi n}}$ is the mean free path for each gate voltage. Here, v_F is the Fermi velocity, which is 1.1×10^6 m/s as measured in epitaxial graphene [4]. Figure S5 shows an overlap of the magnetoresistance and a fitted curve with the above model.

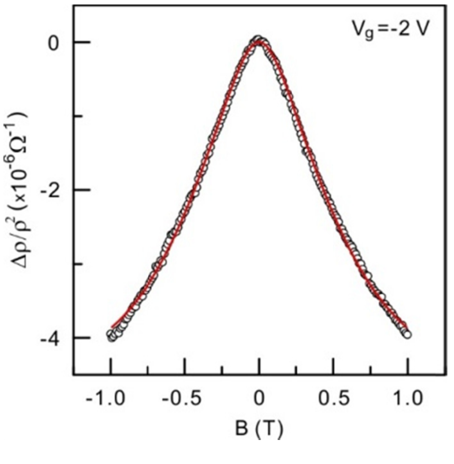

Figure S5. The red line is the fitted curve obtained from the WL model. Circles are the magnetoresistance for V_g= -2 V at 1.9 K.

SI5. Asymmetric electron-hole decoherence: reproduced results

Figure S6 shows the magnetoresistance measurements and extracted decoherence rate as a function of the gate voltage from another device. These results are qualitatively consistent with the presented results leading to the asymmetric electron-hole decoherence described in the main text.

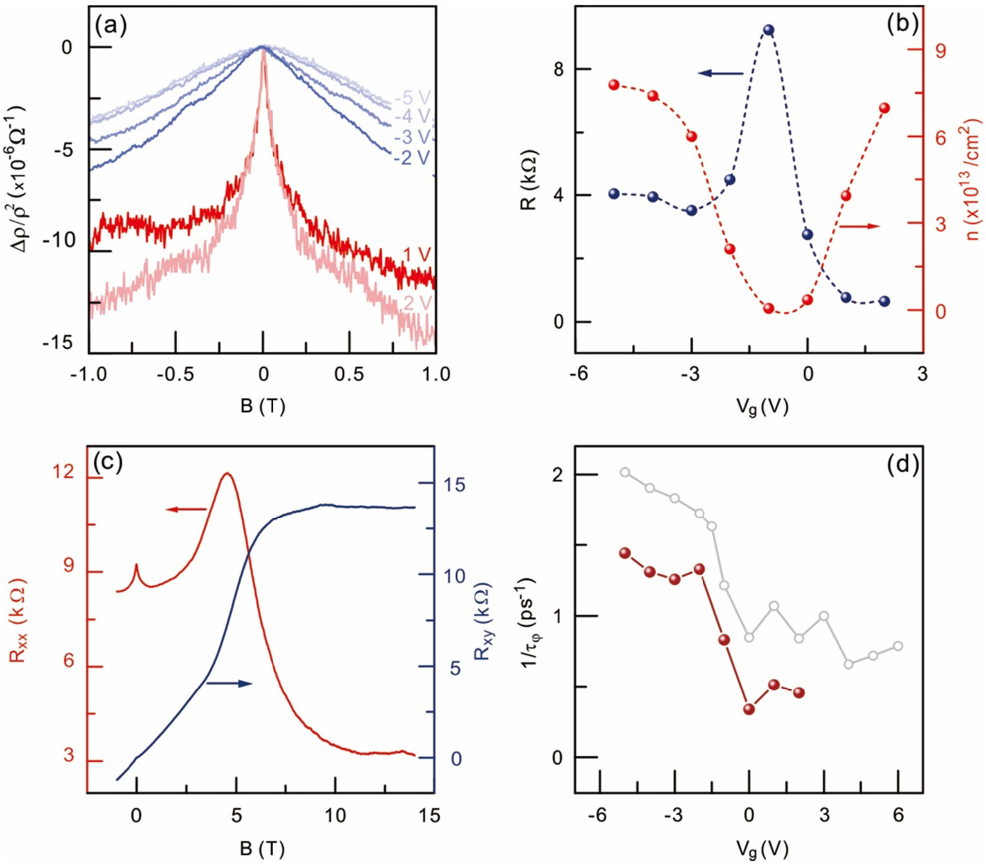

Figure S6. Gate voltage dependence of the magnetoresistance and extracted decoherence rate at 1.9 K. (a) Magnetoresistance for different gate voltages. (b) Gate-voltage dependence of the resistance and induced carrier density. Dotted lines are guides for the eye. (c) Magnetotransport measurement at a gate voltage of -1 V corresponding to 5.42×10^{11} cm^{-2} measured at 1.9 K (d) Red spheres are the extracted decoherence rates from the magnetoresistances for each gate voltage. Gray spheres from the main text are illustrated for comparison.

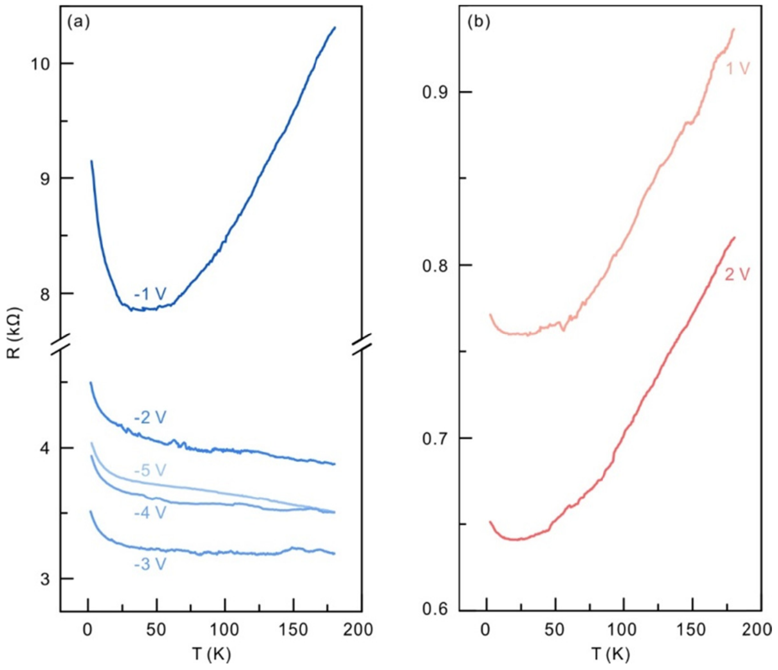

Figure S7. Temperature dependences of the resistivity for different carrier types. (a) Temperature dependence of the resistivity with hole carriers. (b) Temperature dependence of the resistivity for electron carriers.

SI6. Analysis of the temperature dependence of the decoherence rate

We analyze the linear temperature dependence of the decoherence rate in the frame work of dephasing due to inelastic electron-electron scattering[5];

$$\frac{1}{\tau_{ee}} = \alpha \cdot \frac{k_B T}{\hbar} \cdot \frac{\rho e^2}{h} \cdot \ln \frac{h}{2e^2 \rho} \quad (2)$$

$$\frac{1}{\tau_\phi} \approx \frac{1}{\tau_{ee}} + \frac{1}{\tau_o} \quad (3)$$

Figure S8 depicts an example of a fitting to the temperature dependence of the extracted decoherence rate at a gate voltage of -2 V. The determined slope, α and $1/\tau_{ee}$ are approximately 3.5 and 0.14 ps^{-1}, respectively. $1/\tau_o$ is about 1.2 ps^{-1}.

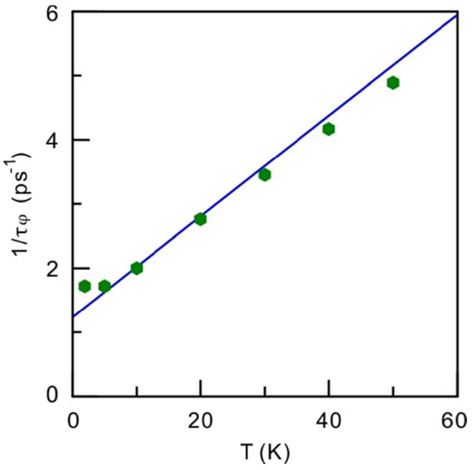

Figure S8. Temperature dependence of the extracted decoherence rate at a gate voltage of -2 V from the device presented in the main text.

References

1. Mattias, K. *et al.* Comeback of epitaxial graphene for electronics: large-area growth of bilayer-free graphene on SiC. *2D Materials* **3**, 041002 (2016).
2. Lee, D. S. *et al.* Raman Spectra of Epitaxial Graphene on SiC and of Epitaxial Graphene Transferred to SiO2. *Nano Lett.* **8**, 4320-4325, doi:10.1021/nl802156w (2008).
3. McCann, E. *et al.* Weak-localization magnetoresistance and valley symmetry in graphene. *Phys. Rev. Lett.* **97**, 146805, doi:10.1103/PhysRevLett.97.146805 (2006).
4. Miller, D. L. *et al.* Observing the Quantization of Zero Mass Carriers in Graphene. *Science* **324**, 924-927, doi:10.1126/science.1171810 (2009).
5. Altshuler, B. L., Aronov, A. G. & Khmelnitsky, D. E. Effects of electron-electron collisions with small energy transfers on quantum localisation. *Journal of Physics C: Solid State Physics* **15**, 7367 (1982).
